# Type A2 BTB Members Decrease the ABA Response during Seed Germination by Affecting the Stability of SnRK2.3 in *Arabidopsis*

**DOI:** 10.3390/ijms21093153

**Published:** 2020-04-30

**Authors:** Guohua Cai, Yuan Wang, Guoqing Tu, Pengwang Chen, Sheng Luan, Wenzhi Lan

**Affiliations:** 1 State Key Laboratory for Pharmaceutical Biotechnology, College of Life Sciences, Nanjing University, Nanjing 210093, China; guohuacai1312@163.com (G.C.); 18205098961@163.com (G.T.); MG1930002@163.com (P.C.); 2 Shanghai Center for Plant Stress Biology, CAS Center for Excellence in Molecular Plant Sciences, Shanghai 201602, China; wangyuan19890715@163.com; 3 Department of Plant and Microbial Biology, University of California, Berkeley, CA 94720, USA

**Keywords:** BTB-A2 protein, seed germination, ABA, SnRK2.3, *Arabidopsis*

## Abstract

The *Arabidopsis* genome comprises eighty genes encoding BTB (broad-complex, tramtrack, and bric-a-brac) family proteins that are characterized with the BTB domain and that potentially serve as substrate adaptors for cullin-based E3-ligases. In addition to the BTB domain, most BTB proteins also contain various other interaction motifs that probably act as target recognition elements. Here, we report three members of the BTB-A2 subfamily that distinctly only contain the BTB domain, BTB-A2.1, BTB-A2.2, and BTB-A2.3, that negatively regulates abscisic acid (ABA) signaling in *Arabidopsis*. *BTB-A2.1*, *BTB-A2.2*, and *BTB-A2.3* encoded cytoplasm- and nucleus-localized proteins and displayed highly overlapping expression patterns in *Arabidopsis* tissues. Disruption of these three genes, but not single or double mutants, resulted in a decrease in ABA-induced inhibition of seed germination. Further analyses demonstrated the expression levels of these three genes were up-regulated by ABA, and their mutation increased ABA signalling. Importantly, protein-protein interaction assays showed that these three BTB-A2 proteins physically interacted with SnRK2.3. Moreover, biochemical and genetic assays indicated that BTB-A2.1, BTB-A2.2, and BTB-A2.3 decreased the stability of SnRK2.3 and attenuated the SnRK2.3 responsible for the ABA hypersensitive phenotype of seed germination. This report thus reveals that BTB-A2s serve as negative regulators for balancing the intensity of ABA signaling during seed germination.

## 1. Introduction

Seed germination is crucial for next-generation plant growth during the life cycle. Seed germination is frequently a consequence of the competitive interaction between the growth potential of embryonic material and the limited mechanical force of its surrounding tissues, of which the process is elaborately regulated by internal and external signals [1,2,3,4]. Several plant hormones are involved in the control of seed germination [5]. Abscisic acid (ABA) is an important hormone which modulates seed dormancy and germination [5]. More specifically, ABA induces seed dormancy and inhibits seed germination. Recently, researchers have comprehensively revealed the integral components of ABA metabolism and ABA signalling. The main pathways of ABA biosynthesis occur both in plastids and in the cytosol and begin from the precursor isopentenyl diphosphate (IPP) [6]. Recent advances in *Arabidopsis* have revealed the core ABA signalling pathway. Without ABA, there is a physical interaction between protein phosphatases 2C (PP2Cs) and sucrose non-fermenting-1-related protein kinases 2 (SnRK2s), which inhibits the phosphorylation activity of SnRK2s and therefore turns ABA signalling off [7]. In the presence of ABA, ABA is perceived and bound by the PYR/PYL receptor family, which leads to conformational changes of the receptor proteins and formation of a platform for physical association with PP2Cs [8,9,10]. The released SnRK2s are then activated and can phosphorylate the downstream proteins to turn on ABA signalling [7,11]. Among those modulators, SnRK2s, as central components, positively modulate ABA signalling, and the regulation of SnRK2 activity is important for switching ABA signalling on or off. Recently, studies that address the turnover of core ABA signalling component SnRK2s have been published. Recent studies have concentrated on the effects of phosphorylation of SnRK2.2/2.3/2.6 at the regulation of the protein level. Brassinosteroid (BR)-insensitive 2 (BIN2) phosphorylates SnRK2.2 and SnRK2.3, enhancing their kinase activity levels [12,13]. ARK, a group B3 Raf-like MAP kinase kinase kinase, is an important signaling component that regulates the activity of SnRK2 in basal land plants such as moss [14]. Casein kinase 2 (CK2) kinase regulates the SnRK2.6/SRK2E/OST1 protein stability [15]. Additionally, protein degradation plays a significant role in modifying the mediator proteins functioning in many biological processes so that plants can appropriately adapt to cellular signals and environmental stimuli. It is reported that AtSCF^AtPP2-B11^ regulates plants to response to ABA by degrading SnRK2.3 [16]. These researchers demonstrated that several components that modify the modulators of ABA signalling may further reveal the molecular foundation of ABA signalling networks, allowing plants to adapt the environment to grow, develop, and reproduce.

Protein turnover in most cellular processes requires a tightly controlled coordination between synthesis and degradation, allowing cells to rapidly adapt to various internal and external cues [17]. Protein ubiquitination modification is an important post-translational regulatory mechanism. Ubiquitin ligases (E3) are multiprotein complexes that mediate the transfer of ubiquitin from ubiquitin binding enzymes E2 to specific substrate proteins [17]. The CUL3-based E3-ligases in animals and plants are assembled with the members of the BTB family through the BTB domain. Thus, BTB proteins potentially act as the substrate adaptors for CUL3-based E3-ligases [17,18]. The family of BTB (Bric-a-brac, Tramtrack and Broad-complex) proteins at the N terminus contain about 120 conserved residues called the BTB domain, which has been widely studied in eukaryotes, based on its wide array of functions such as transcriptional regulation, chromatin organization, cytoskeletal regulation, and protein degradation [19,20,21]. In *Arabidopsis*, there are about 80 putative BTB proteins split into 10 families (A-J), with two families divided further into two subfamilies (A1/A2 and D1/D2), and functions of several members in this family have been identified [22]. For example, *Arabidopsis* ethylene overproducer 1 (ETO1), ETO1-like 1 (EOL1), and ETO1-like 2 (EOL2) are involved in the regulation of ethylene synthesis through the recognition and presumed ubiquitination of ACC synthase 5 (ACS5) and related enzymes [23]. *Arabidopsis* non-expresser of pathogenesis-related genes (NPR1) and NPR3/NPR4 are proposed to be SA (salicylic acid) receptors, but play opposite roles in regulating SA-induced plant immunity [24]. *Arabidopsis* ARIA positively regulates ABA responses possibly through direct interaction with the ABA response transcription factor ABF2 [25].

Most well-documented BTB proteins belong to the members that contain various other interaction motifs besides the BTB domain. However, the functions of other BTB proteins containing only the BTB domain without other interaction motifs, including the BTB-A2 subfamily, are less known. In this study, we characterized three members of the BTB-A2 subfamily, BTB-A2.1 (AT5G41330), BTB-A2.2 (AT3G09030), and BTB-A2.3 (AT2G24240), in *Arabidopsis*. We employed a reverse genetics method to generate and phenotype the multiple knockout lines of *Arabidopsis BTB-A2.1*, *BTB-A2.2*, and *BTB-A2.3* to explore their physiological functions. Our genetic data suggested that cytoplasm- and nucleus-localized BTB-A2s functioned redundantly in ABA-induced inhibition of seed germination. We further demonstrated that these three BTB-A2s physically interacted with SnRK2.3 and decreased the stability of SnRK2.3, leading to a decrease in the SnRK2.3 responsible for seed germination. Thus, this study has revealed a previously unrecognized mechanism of maintaining the appropriate ABA signalling level via SnRK2.3 in finely tuned seed germination.

## 2. Results

### 2.1. Subcellular Location and Expression Pattern of Arabidopsis BTB-A2s

**Figure 1:**
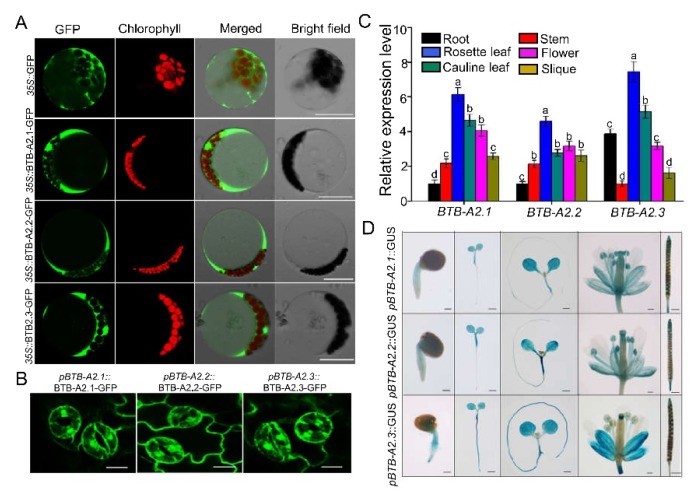
Localization and expression patterns of *Arabidopsis* BTB-A2.1, BTB-A2.2, and BTB-A2.3. (**A**) Subcellular localization of BTB-A2.1, BTB-A2.2, and BTB-A2.3 transiently transformed in *Arabidopsis* protoplasts. Images from top to bottom: GFP empty vector, *35S::*BTB-A2.1-GFP fusion protein, *35S::*BTB-A2.2-GFP fusion protein, and *35S::*BTB-A2.3-GFP fusion protein. Columns from 1 to 4 are GFP fluorescence, chlorophyll autofluorescence, merged images, and bright field, respectively. Bars = 20 μm; (**B**) Localization of BTB-A2.1, BTB-A2.2, and BTB-A2.3 in guard cells of *pBTB-A2.1::BTB-A2.1-GFP*, *pBTB-A2.2::BTB-A2.2-GFP,* and *pBTB-A2.3::BTB-A2.3-GFP* transgenic *Arabidopsis* leaves. Bars = 20 μm; (**C**) Relative expression level of *BTB-A2.1*, *BTB-A2.2*, and *BTB-A2.3* in different tissues of 5-week-old WT plants grown in hydroponic culture. The expression level was detected by qPCR. *ACTIN2* was employed as an internal standard. Data are the mean ±SD. n = 3. Different letters indicate a significant difference (*p* < 0.05); (**D**) Expression patterns of *pBTB-A2.1::GUS*, *pBTB-A2.2::GUS,* and *pBTB-A2.3::GUS* in transformed *Arabidopsis* plants. Columns from 1 to 5 are a seedling at 1 day (bars = 200 µm), a seedling at 3 days (bars = 1 mm); a seedling at 7 days (bars = 1 mm), inflorescence (bars = 100 µm), and fruit pod (bars = 1 mm), respectively.

Unlike most members of the *Arabidopsis* BTB superfamily, which have additional motifs, the clade A2-type BTB proteins (BTB-A2) only have a BTB domain [22]. Phylogenetic analysis and sequence alignment indicated that three genes, At5g41330, At3g09030, and At2g24240, encode products with the closest similarities, and were named BTB-A2.1, BTB-A2.2, and BTB-A2.3, respectively. A BLASTp search was performed to examine putative BTB-A2s homologs in *Arabidopsis*, *Drosophila*, *Homo sapiens,* and *Cricetulus griseus* using these three BTB-A2s members as query sequences. The phylogenetic tree based on the query sequences agreed well with the evolutionary relationship among these species (Figure S1), suggesting BTB proteins are functionally important in eukaryotes. Based on the predicted data from TMHMM analysis (http://www.cbs.dtu.dk/services/TMHMM-2.0/), BTB-A2.1, BTB-A2.2, and BTB-A2.3 do not have a transmembrane domain (Figure S1B), indicating they may be water-soluble proteins. Because the cellular function of a protein primarily depends on its subcellular localization, we generated three constructs that harbored GFP fused to the C-terminus of coding sequences of BTB-A2.1. BTB-A2.2, or BTB-A2,3, driven by the 35S cauliflower mosaic virus promoter, and transferred these constructs into *Arabidopsis* mesophyll protoplasts. The fluorescence signals generated by BTB-A2.1-. BTB-A2.2-, or BTB-A2,3-GFP were all detected in the cytoplasmic areas and did not appear the areas occupied by chloroplasts or the central vacuole, the same distribution as the control GFP signals (Figure 1A), indicating that these three BTB-A2s proteins probably localized in the cytosol. To further confirm the localization, we generated stable expression transgenic plants of BTB-A2-GFP controlled by the promoter fragment of *BTB-A2.1*, *BTB-A2.2*, or *BTB-A2.3*, and found that the GFP signals occurred in the cytoplasm and nucleus of *Arabidopsis* leaf guard cells (Figure 1B). Together, these results indicated that BTB-A2s have overlapping subcellular localization in the cytoplasm and nucleus, consistent with the predicted data from TMHMM analysis.

To identify the plant tissues where BTB-A2s may function in *Arabidopsis*, we performed a real-time quantitative PCR (qPCR) analysis to examine the expression patterns of *AtBTB-A2s* in 5-week-old *Arabidopsis* plants and found that the transcripts of *BTB-A2.1*, *BTB-A2.2*, and *BTB-A2.3* were ubiquitously expressed in all the tissues or organs investigated, with the highest levels in leaves (Figure 1C). In addition, the transcripts of *BTB-A2.1* and *BTB-A2.2* were present at the highest level in the rosette leaves and the lowest in the roots; however, the expression of *BTB-A2.3* showed a different pattern, with the highest level in the rosette leaves and higher level in the roots than flowers, stems and siliques. To characterize the expression patterns of *BTB-A2.1*, *BTB-A2.2*, and *BTB-A2.3* in more detail, we generated transgenic lines harboring the *β*-glucuronidase (GUS) reporter controlled by the promoter fragment of *BTB-A2.1*, *BTB-A2.2*, or *BTB-A2.3* and performed histochemical staining of GUS activity on these lines. As shown in Figure 1D, in the three transgenic lines, the GUS activity showed more active signals in cotyledons than in the roots during the post-germination and vegetative phases. In mature plants, the GUS signals mainly appeared in flowers, in particular anthers, and young siliques, consistent with the qPCR analysis (Figure 1D). Furthermore, the overall expression patterns of *BTB-A2.1*, *BTB-A2.2*, and *BTB-A2.3* were highly similar, suggesting that they have a possible overlap of functions.

### 2.2. AtBTB-A2s Are Involved in ABA-Responsive Germination

To characterize the roles of BTB-A2s in *Arabidopsis* plants, we screened the putative transfer deoxyribonucleic acid (T-DNA) insertion mutants of the *BTB-A2.1*, *BTB-A2.2*, or *BTB-A2.3* gene obtained from the *Arabidopsis* Biological Resource Center (ABRC) and confirmed the positions of gene knockout by PCR analysis and DNA sequencing (Figure S2A). We found the mutant line SALK_114523C, SALK_101331, or CS825825 respectively lacked detectable *BTB-A2.1*, *BTB-A2.2*, or *BTB-A2.3* transcripts (Figure S2B), and thus the mutants were named *btb-a2.1*, *btb-a2.2*, and *btb-a2.3*, respectively, in this study.

Because the three *BTB-A2s* genes have the same subcellular localization and highly overlapping expression patterns, we expected that they may function redundantly. Furthermore, BTB-type proteins have been reported to generally function in heterodimerization or multimerization [26,27]. We thus performed yeast two-hybrid assays to determine the interactions among BTB-A2.1, BTB-A2.2, and BTB-A2.3. Yeast growth assays showed that BTB-A2.1 interacted with BTB-A2.2 and BTB-A2.3, and BTB-A2.3 interacted with itself (Figure S3), suggesting that BTB-A2.1, BTB-A2.2, and BTB-A2.3 may function in the form of a multimer complex. Therefore, we produced double and triple mutants by crossing all the single mutants to analyze possible functional redundancy. The *BTB-A2s* transcripts in produced double and triple mutants were detected by RT-PCR (Figure S2C). Grown in the soil in our laboratory, *btb-a2.1/2/3* triple mutant plants grew in a similar manner as the wild type (WT) plants during the life cycle, including vegetative and reproductive periods (Figure S4).

Multiple BTB-type proteins neighboring BTB-A2s have been reported to be involved in hormone signaling. We initially examined salicylic acid (SA)-related phenotypes in WT and *btb-a2.1/2/3* plants. We applied exogenous SA, benzoic acid (BA), a putative precursor of SA, or *p*-hydroxybenzoic acid (*p*HBA), an isomer of SA [28], to treat WT and *btb-a2.1/2/3* plants and found these two plants displayed a similar root growth phenotype under these treatments (Figure S5A,B). Because several homologs of BTB-A2s have been reported to contribute to ethylene signaling [23], we then evaluated the role of ethylene signaling by the addition of 1-aminocyclopropane-1-carboxylate (ACC), the precursor of ethylene biosynthesis, or Ag^+^, a blocker of ethylene binding with receptors for darkness and light [29]. We found that WT and *btb-a2.1/2/3* displayed comparable hypocotyl and root length grown in darkness and light (Figure S5C–F). These results demonstrated that BTB-A2s might not be involved in SA and ethylene signaling.

**Figure 2:**
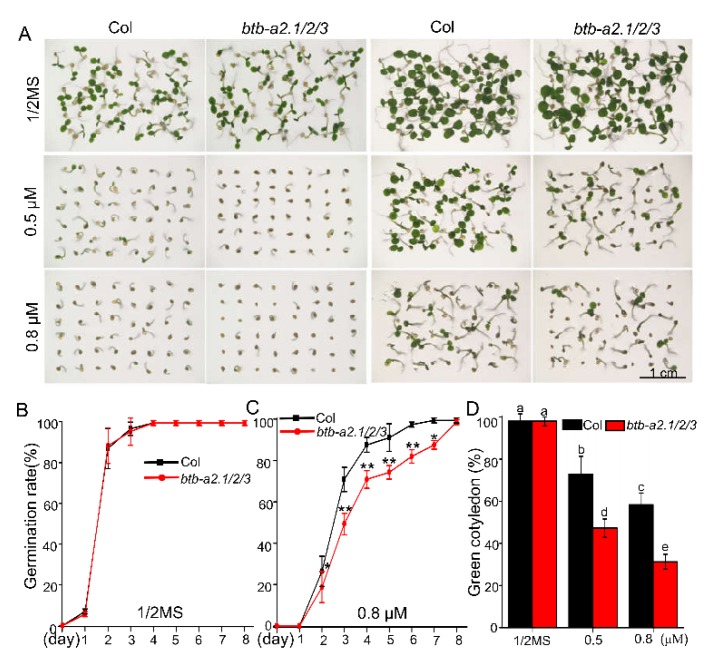
ABA responses of *Arabidopsis btb-a2.1/2/3* during seed germination. (**A**) Germination of WT and *Arabidopsis* triple mutant *btb-a2.1/2/3* in 1/2 MS medium without or with 0.5 μM, 0.8 μM ABA. The images were taken after three days (first two columns) and five days (last two columns) of stratification; (**B**) Germination rate statistics of WT and *btb-a2.1/2/3* under normal conditions; (**C**) Germination rate statistics of WT and *btb-a2.1/2/3* under 0.8 μM ABA conditions. Data are the mean ± SD. Asterisks indicate a significant difference compared with WT (* *p* < 0.05, ** *p* < 0.01, Student’s t-test); (**D**) Green cotyledon statistics of WT and *btb-a2.1/2/3* under normal, 0.5 μM ABA, and 0.8 μM ABA conditions. About 150 seeds of each line were used in each experiment, and each assay was repeated three times. Data are the mean ± SD. Values labeled with different letters are significantly different (*p* < 0.05).

**Figure 3:**
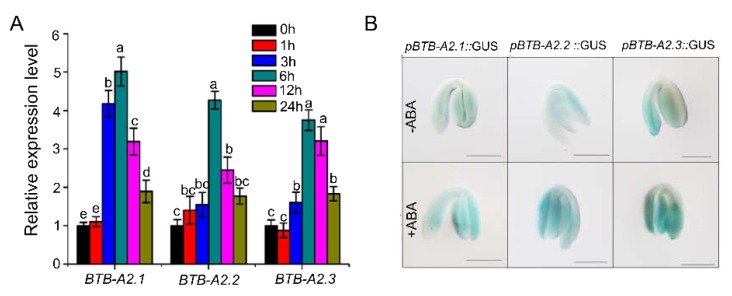
Expression levels of *BTB-A2.1*, *BTB-A2.2*, and *BTB-A2.3* under ABA induction. (**A**) Transcription expression levels of *BTB-A2.1*, *BTB-A2.2*, and *BTB-A2.3* under ABA induction. The expression level was determined by qPCR. The 7-day-old WT seedlings were treated with 50 μM ABA at the specified time point, and samples were collected for RNA extraction. The *ACTIN2* gene was used as an internal reference. Data are the mean ±SD. n = 3. Values labeled with different letters are significantly different (*p* < 0.05); (**B**) GUS staining of one-day-old imbibed *pBTB-A2.1*::GUS, *pBTB-A2.2*::GUS, and *pBTB-A2.3*::GUS transgenic seeds with or without 50 μM ABA for 6 h. Bar = 1 mm.

We noticed that there are several ABA-related *cis*-elements in the promoter region of *BTB-A2.1*, *BTB-A2.2*, or *BTB-A2.3* using *PlantCARE* analysis (Figure S6), suggesting that they may be involved in the ABA response. To test this possibility, we treated freshly harvested seeds of the WT and *btb-a2.1/2/3* triple mutant with ABA to examine whether *BTB-A2*s might function in seed germination. We found that the *btb-a2.1/2/3* triple mutant was more sensitive to ABA than the WT during germination (Figure 2). For example, the germination rate and the green cotyledon rate of the triple mutant were almost similar in comparison to those of the WT on half-strength Murashige and Skoog (MS) medium in the absence of exogenous ABA. However, *btb-a2.1/2/3* displayed a significantly lower germination rate and green cotyledon rate than WT at 0.5 µM and 0.8 µM ABA, indicating that *btb-a2.1/2/3* was more sensitive than WT in ABA-induced inhibition of seed germination. We found that single and double mutants had no significant difference in seed germination without or with exposure to exogenous ABA treatment, taking into account the possible functional redundancy of *BTB-A2s* genes (Figure S7). Based on the finding of the phenotype, we further analyzed the expression levels of *BTB-A2.1*, *BTB-A2.2*, and *BTB-A2.3* in response to ABA. qPCR assays revealed that *BTB-A2.1*, *BTB-A2.2*, and *BTB-A2.3* were all induced by exogenous ABA at the indicated time points in 7-day-old seedlings at the mRNA level (Figure 3A). Considering that ABA as an important hormone inhibits seed germination [5] and the *btb-a2.1/2/3* triple mutant was more sensitive to ABA, we examined the *BTB-A2* expression profiles more precisely during seed germination. The GUS staining analysis showed that *AtBTB-A2s* were expressed in the embryo, and the expression levels of *BTB-A2.1*, *BTB-A2.2*, and *BTB-A2.3* in one day-imbibed seeds accelerated in the presence of ABA (Figure 3B). These observations suggest that BTB-A2.1, BTB-A2.2, and BTB-A2.3 may be negatively and redundantly involved in ABA-induced inhibition of seed germination.

### 2.3. Arabidopsis BTB-A2s May Be Involved in ABA Signalling

The finding that *Arabidopsis btb-a2.1/2/3* showed certain sensitivity under ABA conditions promoted us to explore whether *Arabidopsis* BTB-A2.1, BTB-A2.2, and BTB-A2.3 were involved in the ABA synthesis pathway or ABA signalling during seed germination. We characterized the expression levels of ABA biosynthesis-related genes [6], including *AtABA1*, *AtABA3*, *AtAAO3,* and *AtNCED5* in WT and the *btb-a2.1/2/3* triple mutant with or without ABA treatment. As the qPCR data exhibited, all genes were induced by exogenous ABA, and expression levels of these genes had no apparent difference between WT and the *btb-a2.1/2/3* triple mutant, indicating that BTB-A2.1, BTB-A2.2, and BTB-A2.3 may not be involved in alteration of endogenous ABA level (Figure S8).

**Figure 4:**
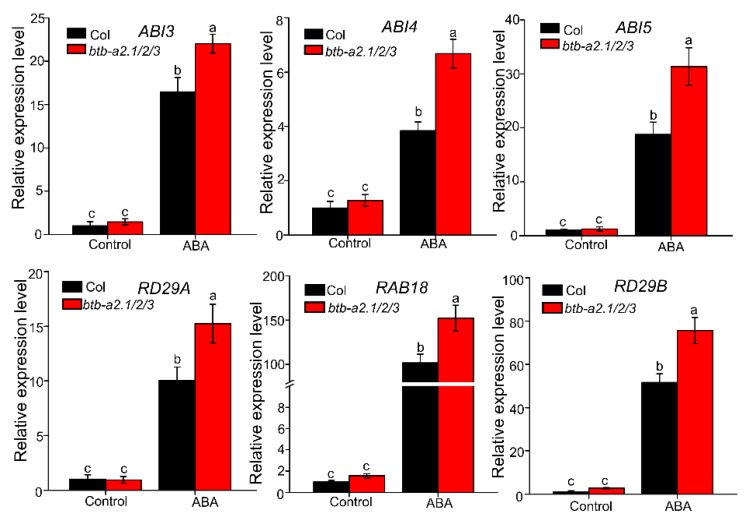
Expression analysis of ABA signaling-related genes. Total RNA was isolated from 7-day-old WT and *btb-a2.1/2/3* seedlings growing under normal and 0.5 μM ABA conditions. The *ACTIN2* gene was used as an internal reference. Data are the mean ± SD. n = 3. Different letters indicate a significant difference (*p* < 0.05).

The expression levels of several well-characterized ABA signal regulator genes (*ABI3*, *ABI4*, *ABI5*, *RAB18*, *RD29A*, *RD29B*) in WT and the *btb-a2.1/2/3* triple mutant were also analyzed. As expected, these genes were induced by exogenous ABA in WT, in agreement with previous studies [30,31,32]. qPCR data showed that these genes were all induced by exogenous ABA, but the expression levels of these genes in the *btb-a2.1/2/3* triple mutant were markedly elevated compared with those in WT plants (Figure 4). These results showed that *Arabidopsis* BTB-A2.1, BTB-A2.2, and BTB-A2.3 might respond to ABA mainly dependening on ABA signalling.

### 2.4. BTB-A2s Physically Interact with SnRK2.3

**Figure 5:**
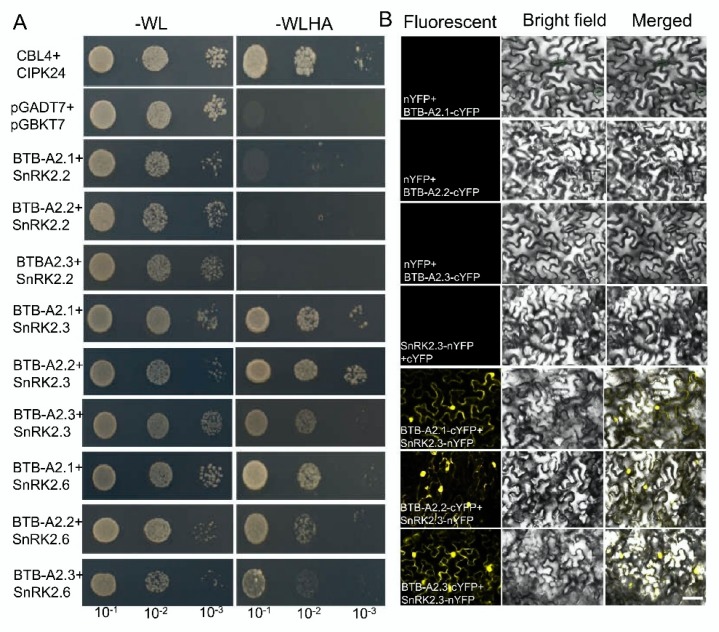
BTB-A2s interact with SnRK2.3. (**A**) The interactions of BTB-A2s with SnRK2.2, SnRK2.3, and SnRK2.6 by yeast two-hybrid assay. Saturated cultures were spotted onto SD-WL and SD-WLHA at different dilutions (10^−1^, 10^−2^, and 10^−3^). The co-transformants of vectors CBL4-AD and CIPK24-BD were employed as positive controls, and co-transformants of the empty vectors pGADT7 and pGBKT7 were employed as negative controls; (**B**) The interactions of BTB-A2s with SnRK2.3 were analyzed using BiFC assay in *N. benthamiana* leaves. Columns from left to right are the fluorescent signal, bright field images, and merged images, respectively. Bar = 50 µm.

Considering that PP2Cs and SnRK2s as the switch turn ABA signalling on or off [11], we explored the relationship between BTB-A2s and the core components of the major ABA signalling pathway. We first employed the yeast two-hybrid system to screen the putative interacting proteins by representative BTB-A2.1, finding that there were no PP2Cs interacting with BTB-A2.1 (Figure S9). Because SnRK2.2, SnRK2.3, and SnRK2.6 share high levels of protein similarity [33], we next validated the interaction between SnRK2s and BTB-A2s. After screening, strong interactions were detected between BTB-A2s and SnRK2.3 and between BTB-A2s and SnRK2.6, whereas no interactions were found between BTB-A2s and SnRK2.2 by yeast two-hybrid assay (Figure 5A). To further confirm the interaction, we detected a strong fluorescent signal in both the nucleus and cytoplasm between BTB-A2s and SnRK2.3, and between BTB-A2s and SnRK2.6, but no fluorescent signal in *Nicotiana benthamiana* leaf cells expressing the negative control constructs by a bimolecular fluorescence complementation (BiFC) assay (Figure 5B and Figure S10). These results demonstrate there exist physical associations between BTB-A2 and SnRK2.3, and between BTB-A2 and SnRK2.6.

### 2.5. BTB-A2s Decrease the Stability of SnRK2.3

**Figure 6:**
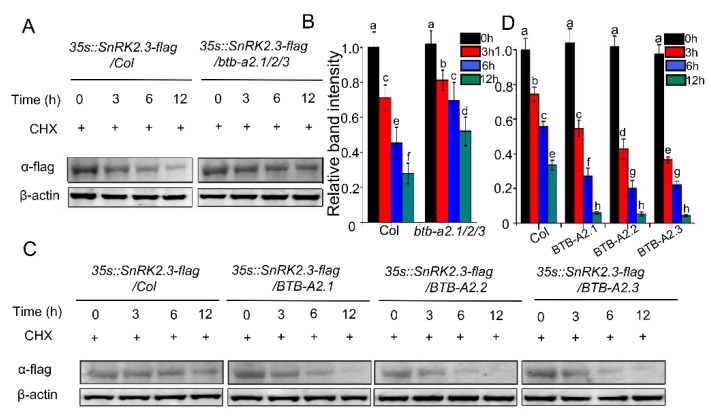
*Arabidopsis* BTB-A2s affect the stability of SnRK2.3. (**A**,**C**) The content of SnRK2.3 in WT, triple mutant *btb-a2.1/2/3* plants, and *BTB-A2s*-OE plants was detected. Seven-old-day WT, triple mutant *btb-a2.1/2/3*, and *BTB-A2s*-OE seedlings were subjected to 50 μM CHX (a protein synthesis inhibitor), and total plant proteins were extracted at specified time points. The protein level of SnRK2.3 was detected by monoclonal anti-flag antibody. Monoclonal β-actin antibody was used to normalize the loadings; (**B**,**D**) Quantitative analysis of the relative strength of bands based on Image J. Values labeled with different letters indicate a significant difference (*p* < 0.05).

*Arabidopsis* BTB-A2.1, BTB-A2.2, and BTB-A2.3 have the core structure of the BTB domain and protein–protein interaction domain, which may act as the substrate adaptors for CUL3-based E3-ligases [22]. Previous research has reported that E3 ubiquitin ligases affect the degradation/stability of SnRK2s [16]. After the finding of the interaction of *Arabidopsis* BTB-A2.1, BTB-A2.2, and BTB-A2.3 with SnRK2.3 in plant cells, we speculated whether BTB-A2.1, BTB-A2.2, and BTB-A2.3 are involved in ABA signalling by mediating the stability of SnRK2.3. We constructed *35S::SnRK2.3-Flag* transgenic plants in WT and the triple mutant *btb-a2.1/2/3* background, respectively. Seven-day-old transgenic seedlings were treated with 50 µM cycloheximide (CHX, protein biosynthesis inhibitor) for the specified point-in-time, and then proteins were extracted. The SnRK2.3 content was detected with flag antibody. Western blotting showed that the SnRK2.3 protein level decreased more slowly in the *btb-a2.1/2/3* mutant than that in WT at the corresponding time (Figure 6A,B). To further determine the effect of BTB-A2s on the stability of SnRK2.3, we first constructed *35S::BTB-A2.1* overexpression (*BTB-A2.1*-OE), *35S::BTB-A2.2* overexpression (*BTB-A2.2-*OE), and *35S::BTB-A2.3* overexpression (*BTB-A2.3-*OE) transgenic lines. Those *BTB-A2s-*OE transgenic lines that displayed the highest *BTB-A2s* expression levels were selected for further analysis (Figure S11). We then generated transgenic plants of *35S::SnRK2.3-Flag* in WT and the *BTB-A2s*-OE line background, respectively. In the same way, 7-day-old transgenic seedlings were treated with 50 µM CHX for the specified point-in-time, and then proteins were extracted. The western blotting displayed that the SnRK2.3 protein level decreased faster in *BTB-A2s*-OE lines than in the WT at the corresponding times (Figure 6C,D). These results demonstrate that BTB-A2s affect the stability of SnRK2.3 in *Arabidopsis*.

### 2.6. Overexpression of BTB-A2s Attenuates SnRK2.3 Overexpression Lines to the ABA-Hypersensitive Phenotype of Seed Germination 

**Figure 7:**
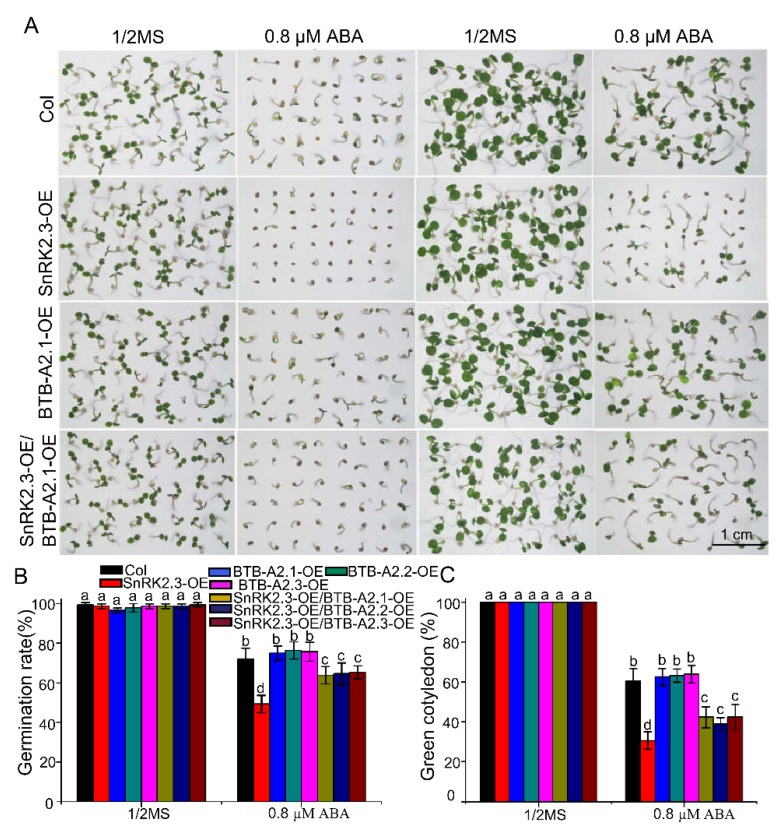
Overexpression of *BTB-A2.1* inhibits the ABA-hypersensitive phenotypes of lines overexpressing *SnRK2.3*. (**A**) The seeds of the WT, *SnRK2.3*-OE line, *BTB-A2.1*-OE line, and *SnRK2.3*-OE line in the *BTB-A2.1*-OE background were germinated in 1/2 MS medium with or without 0.8 μM ABA. The images were taken after three days (first two columns) and five days (last two columns) of stratification; (**B**) The statistics of the germination rate; (**C**) The statistics of the green cotyledon rate. About 150 seeds of each line were used in each experiment, and each assay was repeated three times. Values labeled with different letters indicate a significant difference (*p* < 0.05).

Having ascertained that BTB-A2.1, BTB-A2.2, and BTB-A2.3 physically interacted with SnRK2.3 and affected the stability of SnRK2.3, we predicted that they might affect the physiological function of SnRK2.3, a protein kinase that activates ABA signaling, and we determined whether they genetically negatively modulated SnRK2.3 in ABA-induced inhibition of seed germination. To assess this possibility, we found that *SnRK2.3*-OE lines displayed hypersensitive phenotypes in response to ABA in germination (Figure 7). As BTB-A2s proteins could affect the stability of SnRK2.3, we hypothesized that the overexpression of *BTB-A2s* possibly attenuated the ABA hypersensitivity phenotype of lines overexpressing *SnRK2.3*. Therefore, we performed the seed germination phenotype assay of the Col-0, *SnRK2.3*-OE, *BTB-A2*-OE, and *SnRK2.3*-OE*/BTB-A2*-OE lines. Without the existence of ABA, the germination rates and greening rates did not show significantly visible phenotypic changes among the Col-0, *BTB-A2.1*-OE, *SnRK2.3*-OE, and *SnRK2.3*-OE/*BTB-A2.1*-OE lines (Figure 7). In the presence of 0.8 µM ABA, the germination rates of WT and *BTB-A2.1*-OE seeds were about 72% and 75%, respectively, and the germination rate of *SnRK2.3*-OE seeds was about 49%. Interestingly, the germination rate of *SnRK2.3*-OE*/BTB-A2.1*-OE seeds reached about 64% after three days of stratification (Figure 7B). The green cotyledon rates of the WT and *BTB-A2.1*-OE lines were 60% and 62%, respectively, and the green cotyledon rate of the *SnRK2.3*-OE lines was about 29%. However, the green cotyledon rate of the *SnRK2.3*-OE*/BTB-A2.1*-OE lines reached about 42% after five days of stratification (Figure 7C). The fact that the germination and green cotyledon rates of the *SnRK2.3*-OE*/BTB-A2.1*-OE double overexpression lines were much higher than those of the *SnRK2.3*-OE lines indicated that BTB-A2.1 could decrease the content of SnRK2.3 to alleviate ABA-induced inhibition of seed germination. Likewise, BTB-A2.2 and BTB-A2.3 could also decrease the level of SnRK2.3 to attenuate the ABA-induced inhibition of seed germination. (Figure S12). These findings demonstrate that BTB-A2.1, BTB-A2.2, and BTB-A2.3 may serve as negative regulators of the SnRK2.3 protein to regulate ABA signalling during seed germination.

## 3. Discussion

Post-translational modification (PTM) is the basis of the diverse aspects of eukaryotic cell regulation by precise modulation of the stability of short-lived and abnormal intracellular proteins and also by the modulation of phytohormone signalling by affecting protein activity, localization, assembly, and interaction ability [34]. The ubiquitin-proteasome system (UPS) is a major mechanism underlying the degradation of the specific substrates and maintenance of protein homeostasis in eukaryotes [35,36,37]. Many members of the *Arabidopsis* BTB protein family, which contain the highly conserved BTB domain, have been identified to mediate substrate recognition and recruit the substrate to the Cul3 E3 ubiquitin ligase complex [21,38,39]. In this study, three members of an *Arabidopsis* BTB-A2 subfamily were identified as new negative components that regulated the ABA signaling pathway during seed germination by affecting the stability of SnRK2.3. This finding has not only uncovered a novel physiological function of BTB-A2s in *Arabidopsis* but has also improved our understanding of the mechanism mediating ABA signalling by SnRK2.3 during seed germination.

*Arabidopsis* BTB-A2.1, BTB-A2.2, and BTB-A2.3 localized in both the cytoplasm and nucleus (Figure 1). Considering that these three proteins have the same localization and simple expression patterns, as well as the physical interaction among them, it is reasonable to speculate that they may form a polymer and redundantly participate in biological functions. Phenotype screening found that the triple mutant *btb-a2.1/2/3* was more sensitive to ABA than WT plants during seed germination upon ABA treatment (Figure 2). Moreover, the expression levels of *BTB-A2.1*, *BTB-A2.2*, and *BTB-A2.3* were induced by ABA at the transcript level, especially during seed germination (Figure 3). These findings further ascertain that BTB-A2s are responsible for ABA responses during seed germination. Obviously, BTB-A2.1, BTB-A2.2, and BTB-A2.3 are necessary for appropriate response to ABA in *Arabidopsis* plants, which may contribute to plants to reset the ABA signaling. 

Our functional analyses demonstrated that *Arabidopsis* BTB-A2s play an important role in ABA-induced inhibition of seed germination. Moreover, BTB-A2 functioned in the seed germination process dependent on ABA signalling (Figure 4). Intriguingly, we successfully screened out the substrate receptors for SnRK2.3 turnover, which is pivotal to weaken ABA signalling and ABA-dependent plant growth arrest. Although we found a physical interaction between BTB-A2.1, BTB-A2.2, or BTB-A2.3 with SnRK2.3 and SnRK2.6, but not SnRK2.2 (Figure 5 and Figure S10), it remains unknown why such highly conserved SnRK2s have different binding specificities for BTB-A2s. Furthermore, the *SnRK2.3*-OE plants were more sensitive to ABA than WT plants during seed germination (Figure 7). BTB-A2.1, BTB-A2.2, and BTB-A2.3 act antagonistically with SnRK2.3 to mediate ABA responses during seed germination. The germinating seeds of *BTB-A2*-OE plants displayed subtle insensitivity to ABA compared with WT, which is consistent with the *snrk2.3* mutant exhibiting subtle phenotypes of its ABA response. This is because SnRK2.2, SnRK2.3, and SnRK2.6 have functional redundancy in regulating seed germination [40,41]. *BTB-A2* overexpression alleviated the ABA hypersensitive phenotypes of lines overexpressing *SnRK2.3*. However, the *SnRK2.3*-OE*/BTB-A2s*-OE lines could not completely attenuate the hypersensitive phenotypes of the *SnRK2.3*-OE lines, which indicated that BTB-A2s may function with other proteins to mediate ABA signalling in seed germination or there may be other proteins involved in ABA signalling in seed germination. Overall, these results propose a possibility that BTB-A2s may be involved in the negative feedback regulation of ABA signalling. ABA increased the expression of *BTB-A2*, which in turn negatively modulated the SnRK2.3 stability. Resetting of ABA signalling also requires SnRK2.3 degradation to avoid excessive ABA-induced accumulation of SnRK2.3. This may contribute to attenuate the excessive inhibition of germination processes in plants under the influence of ABA, therefore providing a process to fine-tune ABA signalling. Mechanistic investigations indicated that the modification of SnRK2.3 in ABA signaling is tightly modulated at the post-translational level [34]. Previous research focused on the phosphorylation of SnRK2.2/2.3/2.6 and proteasome-mediated protein degradation of SnRK2.2/2.3/2.6 kinases, as the F-box protein AtPP2-B11 modulates ABA signalling by facilitating SnRK2.3 degradation in *Arabidopsis* [16]. Moreover, we found that *Arabidopsis* BTB-A2s decreased ABA signalling during seed germination by affecting the stability of SnRK2.3, which may enrich the regulatory network of ABA signalling. Further research is needed to identify important modulators that associate with SnRK2s and to determine the regulatory mechanism.

**Figure 8:**
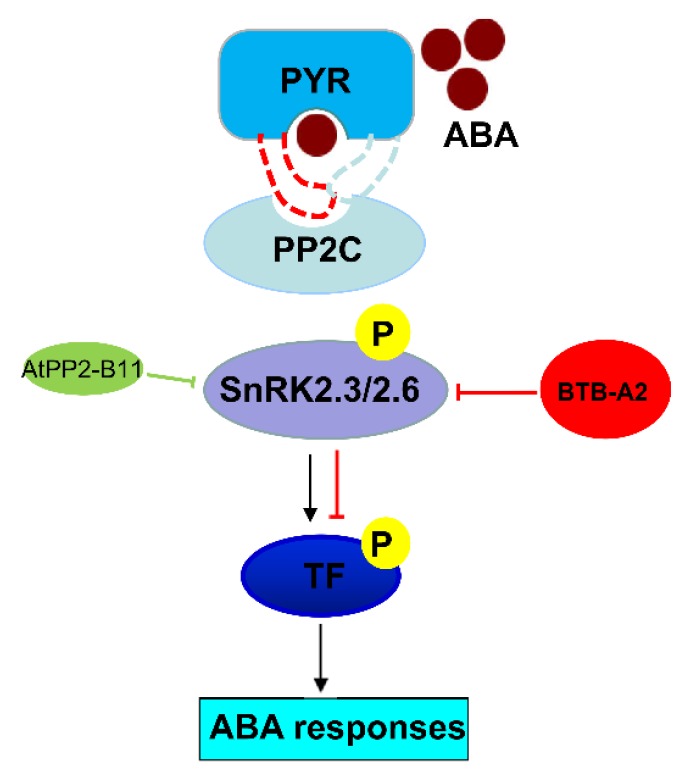
A propose model of *Arabidopsis* BTB-A2s regulating ABA signaling.

In *Arabidopsis*, the BTB protein family contains about 80 members. Although much progress has been successively made in recent years, further research is required to identify the functions of other unknown members to elucidate the biological significance in plants. Collectively, our findings provide genetic and physiological evidence that *Arabidopsis* BTB-A2.1, BTB-A2.2, and BTB-A2.3, which localize in the cytoplasm and nucleus, may act as negative regulators of ABA signalling by impacting SnRK2.3 stability and subsequently weakening the expression of ABA-responsive genes (Figure 8). With gradual identification of negative regulators, such as BTB-A2s, elucidation of the regulatory mechanism will contribute to a better comprehension of ABA signalling. Based on the expression pattern of BTB-A2s in the guard cells of leaves, and the interaction between BTB-A2s and SnRK2.6, it would be interesting to investigate whether BTB-A2.1, BTB-A2.2, and BTB-A2.3 are involved in plant responses to ABA-mediated abiotic and biotic stresses in the future.

## 4. Materials and Methods

### 4.1. Plant Materials and Growth Conditions

*Arabidopsis thaliana* wild type (WT, ecotype Columbia-0) seedlings were used in the research. The T-DNA insertion mutants AT5G41330 (SALK_114523C), AT3G09030 (SALK_101331), and AT2G24240 (CS825825) were obtained from the *Arabidopsis* Biological Resource Center. The positions of T-DNA insertion sites are shown in supporting information Figure S2A. Homozygous mutant plants were screened and identified by PCR using the primers listed in Table S1. Lines of double and triple mutants were constructed by genetic crosses.

For on-plate growth assays, the *Arabidopsis* seeds were sterilized with 75% ethanol for 5 min, washed three times with sterilized water and then sown on 1/2 MS supplement with 1% (*w*/*v*) sucrose and 0.8% (*w*/*v*) phytoblend agar (Caisson Labs, Smithfield, UT, USA). The pH was 5.8. The seeds were stratified at 4 °C for two days and were then placed in a growth chamber (16-h illumination of 150 μmol/m^2^/s, and 8-h dark cycle) at 22 °C. For soil culture, 10-day-old seedlings on 1/2 MS were transferred to nutrient-rich soil (Pindstrup Mosebrug, Denmark) and then grown in a greenhouse with a long-day cycle (16-h illumination of 150 μmol/m^2^/s, and 8-h dark cycle) at 22 °C.

### 4.2. Plasmid Constructs and Generation of Transgenic Plants

To generate *BTB-A2.1*, *BTB-A2.2*, and *BTB-A2.3* overexpression transgenic plants, the full-length coding sequence (CDS) of *BTB-A2.1*, *BTB-A2.2*, *BTB-A2.3* was amplified from the cDNA of WT and then introduced into the binary vector pCAMBIA-3301 under the control of the *CaMV35S* promoter. To generate the *proBTB-A2.1::BTB-A2.1-GFP* construct, we fused the GFP and NOS terminator sequence with the 3609-bp genomic DNA (stop codon was deleted) of *BTB-A2.1*, including the 2232-bp promotor sequence and the 1377-bp coding sequence, and then inserted this recombinant DNA into the binary vector pCAMBIA-1300. The *pBTB-A2.2::BTB-A2.2-GFP*, *pBTB-A2.3::BTB-A2.3-GFP* constructs were obtained using the same method mentioned above. To generate the *pBTB-A2.1*::*GUS* construct, we amplified the promoter of *BTB-A2.1* from WT genomic DNA and introduced this into the modified binary vector pCAMBIA-1300 harboring the GUS reporter gene. *pBTB-A2.2::GUS*, *pBTB-A2.3::GUS* constructs were obtained using the same method mentioned above. To generate *SnRK2.3*-OE transgenic plants, the full-length CDS of *SnRK2.3* was amplified from the cDNA of WT and then cloned into the binary vector pCAMBIA-1302 under the control of the CaMV35S promoter. Those constructs were introduced into the *Agrobacterium tumefaciens* GV3101 strain to transform into *Arabidopsis* plants using the floral dipping approach [42]. The primers used are listed in Table S1.

### 4.3. RNA Isolation, RT-PCR, and qPCR Analysis

Total RNA was extracted from plant samples using TRIzol reagent (Invitrogen, Carlsbad, CA, USA) according to the manufacturer’s instruction. First-strand cDNA synthesis was performed using M-MLV Reverse Transcriptase (Promega, Madison, WI, USA) according to the manufacturer’s instruction. The RT-PCR analysis of gene expression using the cDNA was followed by 27 cycles of PCR. qPCR was performed using an SYBR Green I Master kit (Roche Diagnostics, Hong Kong) according to the manufacturer’s instructions on a CFX Connect Real-Time System (Bio-Rad, Berkeley, CA, USA). *ACTIN2* (AT3G18780) was used as the internal standard in both RT-PCR and qPCR analyses. All individual reactions were performed in triplicate. All gene-specific primers used are listed in Table S1.

### 4.4. Subcellular Localization

For the subcellular localization analysis, the full-length CDS of *BTB-A2.1* was inserted into the pEZS-NL-GFP, which generated a C-terminal fusion with the GFP gene controlled by the CaMV35S promoter. The stop codon was deleted. *35S::BTB-A2.2-GFP* and *35S::BTB-A2.3-GFP* constructs were obtained using the same method mentioned above. The primers used are listed in Table S1.

Isolation and transient expression in *Arabidopsis* protoplasts were conducted according to a published protocol [43]. Imaging was performed on a confocal microscope (LSM-710, Zeiss, Oberkochen, Germany) installed with an argon/krypton laser.

### 4.5. GUS Histochemical Analysis

The GUS staining was performed according to a published protocol [44]. Briefly, the samples were incubated in GUS staining buffer (0.1 mM K3[Fe(CN)6], 5 mM K4[Fe(CN)6], 100 mM Na3PO4, 10 mM EDTA and 0.1% [vol/vol] Triton X-100, pH 7.0), supplemented with 0.5 mM 5-bromo-4-chloro-3-indolyl-β-D-glucuronide, and were vacuum-infiltrated for 15 min. Then, they were placed at 37 °C in darkness for 12 h. The staining solution was removed. The plant tissues were sufficiently destained with 75% (vol/vol) ethanol and were photographed with a microscope (SZX12, Olympus, Tokyo, Japan) installed with a camera.

### 4.6. Yeast Two-Hybrid Assay

Full-length CDS of *BTB-A2.1*, *BTB-A2.2*, *BTB-A2.3* or *SnRK2s* (*SnRK2.2*, *SnRK2.3,* and *SnRK2.6*) was inserted into pGADT7 (AD) and pGBKT7 (BD). The fusion AD and BD constructs were co-transformed into yeast strain AH109 using the lithium acetate transformation method [45]. The transformants (5 μL each) were incubated in synthetic dropout (SD) medium without Trp and Leu (SD-WL) or Trp, Leu, His, and Ade (SD-WLHA) at different dilutions (10^−1^, 10^−2^, and 10^−3^) at 30 °C for 3–5 days. The primers used are listed in Table S1.

### 4.7. BiFC Assay

Full-length CDS of *BTB-A2.1*, *BTB-A2.2*, or *BTB-A2.3* was fused in-frame to the C-terminus of YFP to form *BTB-A2.1-cYFP*, *BTB-A2.2-cYFP*, and *BTB-A2.3-cYFP*. The full-length CDS of *SnRK2.3* and *SnRK2.6* was cloned in-frame into the N-terminus of YFP to generate *SnRK2.3-nYFP* and *SnRK2.6-nYFP*, respectively. All of the constructs were transformed into *A. tumefaciens* strain GV3101 and then infiltrated into *Nicotiana benthamiana* leaves following the method reported by Hu et al. [46]. Infected leaves were examined at 48–72 h after infiltration by a confocal microscope (LSM-710, Zeiss, Oberkochen, Germany). The primers used are listed in Table S1.

### 4.8. Protein Isolation and Immunoblot Analysis

Seven-day-old *Arabidopsis* seedlings were treated with CHX at the indicated times. Seedlings were harvested and frozen in liquid nitrogen for protein extraction, which was performed as described [47]. For immunoblot analysis, total protein extracts were separated on 12% SDS-PAGE and transferred to PVDF membranes. The membrane was blocked in 5% nonfat milk PBST buffer for 2 h at room temperature, and then the membrane was washed by PBST buffer several times. The blot was incubated with specific primary antibodies at 1:1000 dilution in PBST buffer for 1.5 h. After the membrane was washed by PBST buffer several times, the blot was incubated with horseradish peroxidase-conjugated secondary antibody (goat anti-mouse antibody, KW, China) as the secondary antibody at 1:5000 dilution in the same buffer for 1.5 h. The membrane was washed by PBST buffer several times. The immunoblot signal was detected using a Super Signal West Pico Trial kit (Thermo Scientific, Waltham, MA, USA). Blotting of β-actin antibody were performed for protein visualization as a loading control. Quantitative analysis of the band intensity was analyzeda using Image J (National Institutes of Health, Bethesda, MD, USA).

### 4.9. Statistical Analysis

For all experiments, data were analyzed using Excel and Origin 8. Mean values ± SD of at least three replicates are presented. Data were subjected to statistical analyses using Student’s *t*-test (* *p* < 0.05 and ** *p* < 0.01) or one-way analysis of variance (ANOVA) followed by Duncan’s multiple range test (*p* < 0.05).

## Supplementary Materials

The following are available online at https://www.mdpi.com/1422-0067/21/9/3153/s1, Figure S1: Sequence alignment of representative BTB proteins in animals and plants and transmembrane prediction of *Arabidopsis* BTB-A2.1, BTB-A2.2, and BTB-A2.3., Figure S2: Identification of *btb-a2* single mutants, double mutants and triple mutant. Figure S3: *Arabidopsis* BTB-A2s may function in polycomplex. Figure S4: The growth situation of *Arabidopsis btb-a2.1/2/3* in normal condition at each growth stage. Figure S5: *Arabidopsis btb-a2.1/2/3* displayed no different performance compared with WT in SA and ethylene conditions. Figure S6: Analysis of *cis*-elements in the promoter of *AtBTB-A2s*. Figure S7: *Arabidopsis btb-a2* single and double mutant display no sensitivity to ABA in germination. Figure S8: Expression levels of ABA synthesis related genes in WT and triple mutant *btb-a2.1/2/3*. Figure S9: BTB-A2.1 may do not interact with PP2Cs. Figure S10: The interactions between BTB-A2.1, BTB-A2.2, and BTB-A2.3 with SnRK2.6 by BiFC assays in *N. benthamiana* leaves. Figure S11: Expression levels of *BTB-A2.1*, *BTB-A2.2* and *BTB-A2.3 in transformed Arabidopsis* plants by qPCR. Figure S12: Overexpression of *BTB-A2.2*, *BTB-A2.3* inhibits the ABA hypersensitive phenotypes of lines overexpressing *SnRK2.3*. Table S1: Primers used in this study.

## Abbreviations

ABA	Abscisic acid
ACC	1-aminocyclopropane-1-carboxylic acid
ACS5	ACC synthase 5
BiFC	bimolecular fluorescence complementation
BIN2	brassinosteroid (BR)-insensitive 2
BTB	Bric-a-brac (Bab), Tramtrack (Ttk) and Broad-complex (BR-C)
CHX	cycloheximide
CK2	casein kinase 2
ETO1	ethylene overproducer 1
EOL1	ETO1-like 1
EOL2	ETO1-like 2
MS	Murashige and Skoog
NPR1/3/4	non-expresser of pathogenesis-related genes 1/3/4
PP2C	protein phosphatase 2C
PTM	post-translational modification
qPCR	quantitative real-time PCR
SnRK2	sucrose non-fermenting-1-related protein kinase 2
