# Supplementary material for: Type A2 BTB Members Decrease the ABA Response during Seed Germination by Affecting the Stability of SnRK2.3 in Arabidopsis
Source: Int J Mol Sci. 2020 Apr 30;21(9):3153. doi: 10.3390/ijms21093153 (PMC7246803; doi:10.3390/ijms21093153)
Supplement: Supplementary file 1 [file ijms-21-03153-s001.pdf]

Supplementary file

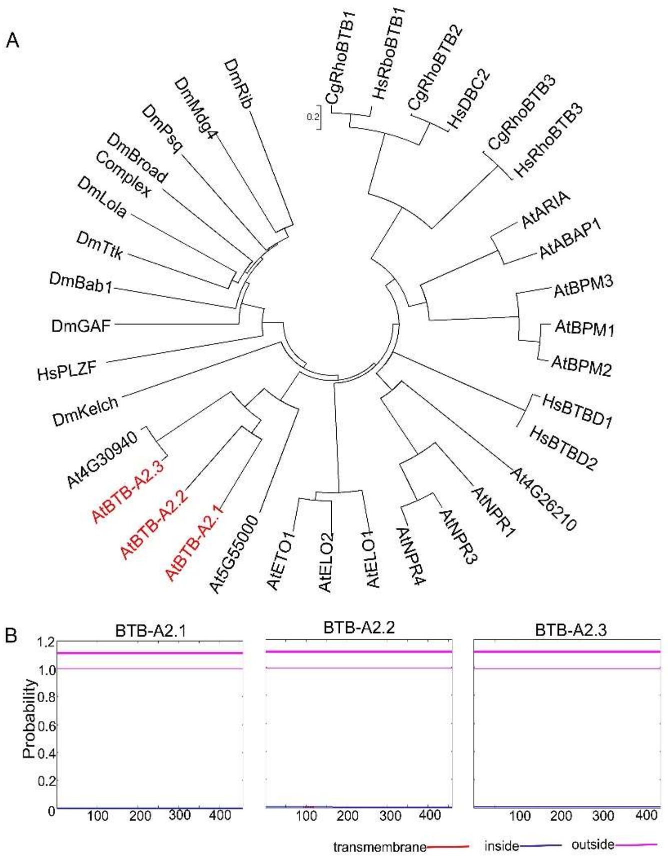

Figure S1. Sequence alignment of representative BTB proteins in animals and plants and transmembrane prediction of *Arabidopsis* BTB-A2.1, BTB-A2.2, and BTB-A2.3. (**A**) Phylogenetic relationship of representative BTB-A2 proteins in animals and plants. DmRib (NP_001261084), DmMdg4 (NP_788698), DmPsq (NP_523686), DmBroad Complex (NP_726750), DmLola (NP_788312), DmTtk (P17789), DmBab1 (NP_728565), DmGAF (Q08605), HsPLZF (NP_001018011), DmKelch (NP_724095), AtETO1 (At3g51770), AtEOL2 (At5g58550), AtEOL1 (At4g02680), AtNPR1 (At1g64280), AtNPR2 (At4g26120), AtNPR3 (At5g45110), AtNPR4 (At4g19660), AtARIA (At5g19330), AtABAP1 (At5g13060), AtBTB-A2.1 (At5g41330), AtBTB-A2.2 (At3g09030), AtBTB-A2.3 (At2g24240), AtBPM1 (AT5G19000), AtBPM2 (At3g06190), AtBPM3 (At2g39760), HsRhoBTB3 (NP055714), HsDBC2 (NP003400), HsRboBTB1 (AAH41791), HsBTBD1 (NP_079514), HsBTBD2 (NP_001011885), CgRhoBTB1 (RLQ76268), CgRhoBTB2 (RLQ55967), CgRhoBTB3 (RLQ73570). (**B**) The transmembrane prediction of *Arabidopsis* BTB-A2.1, BTB-A2.2, and BTB-A2.3.

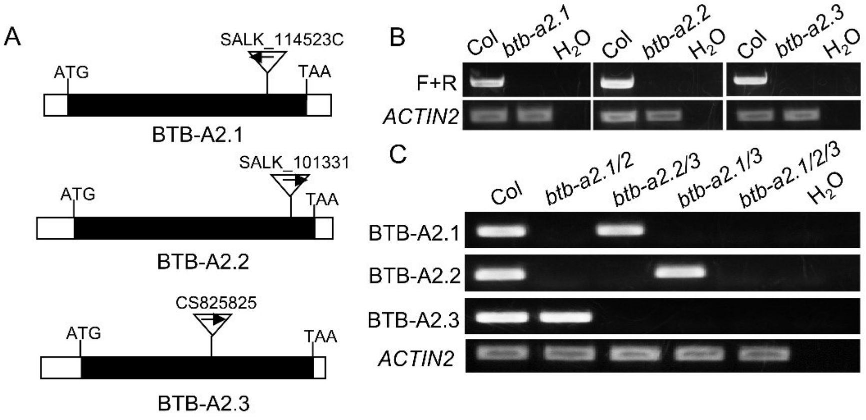

Figure S2. Identification of *btb-a2* single mutants, double mutants and triple mutant. (**A**) Schematic diagrams of gene structure and T-DNA insertion positions of *BTB-A2s*. The 5′ and 3′ non-translation regions were indicated by the hollow frame represents. The exons were indicated by the black solid frame. The position of T-DNA insertion in the mutant was indicated by the triangle. The direction of T-DNA insertions in the mutant was indicated by arrows. (**B**) RT-PCR analysis of the accumulation of *BTB-A2.1*, *BTB-A2.2*, and *BTB-A2.3* transcripts in Col, *btb-a2.1*, *btb-a2.2*, and *btb-a2.3*, respectively. (**C**) RT-PCR analysis of double and triple mutants. Expression of *ACTIN2* gene was used as internal reference.

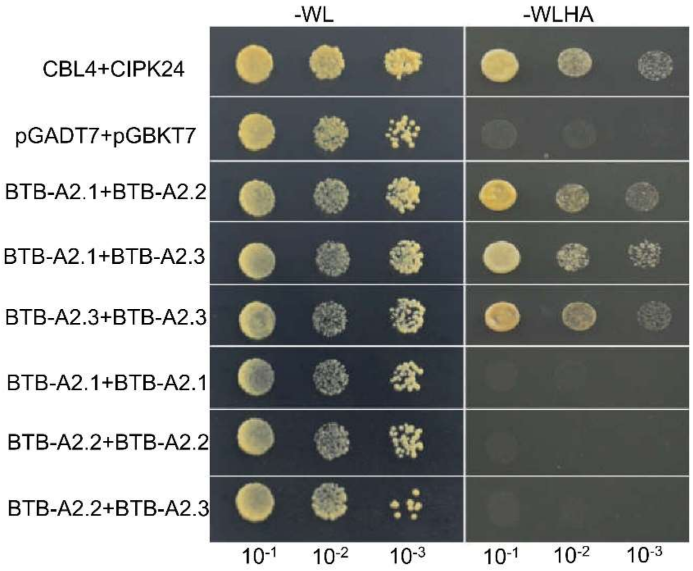

Figure S3. *Arabidopsis* BTB-A2s may function in polycomplex. The interaction among BTB-A2.1, BTB-A2.2 and BTB-A2.3 by yeast two-hybrid assays. Saturated cultures were spotted onto on SD-WL and SD-WLHA at different dilutions (10^{-1}, 10^{-2}, and 10^{-3}).

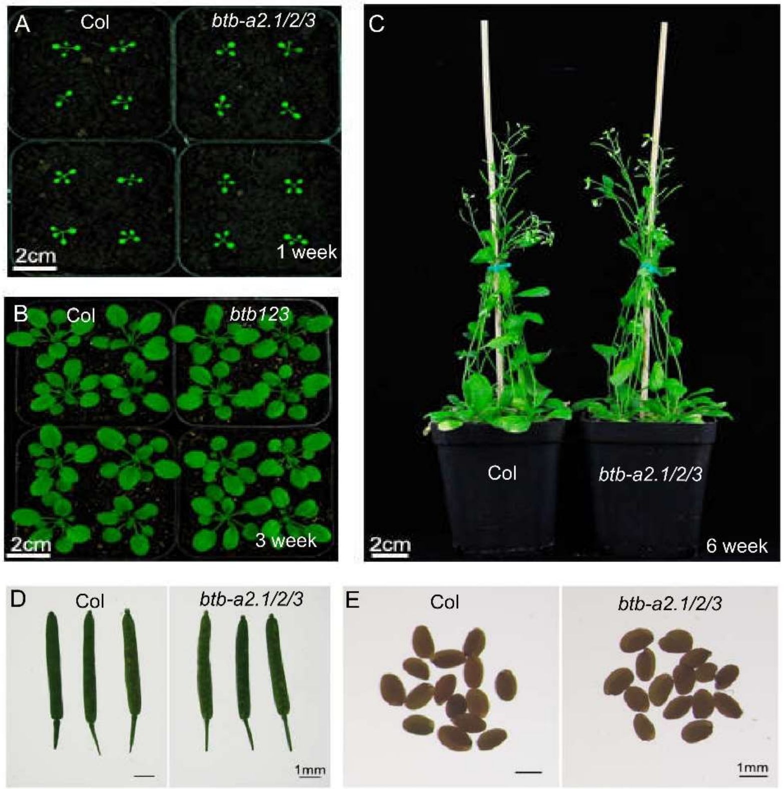

Figure S4. The growth situation of *Arabidopsis btb-a2.1/2/3* in normal condition at each growth stage. (**A-C**) The growth of WT and *Arabidopsis* triple mutant *btb-a2.1/2/3* at one week (**A**), three weeks (**B**), six weeks (**C**), Bar=2cm. (**D**, **E**) The size of siliques and seeds of WT and *Arabidopsis* triple mutant *btb-a2.1/2/3*. Bar=1mm.

Figure S5. *Arabidopsis btb-a2.1/2/3* displayed no different performance compared with WT in SA and ethylene conditions. (**A**) The phenotypic analysis of WT and triple mutant *btb-a2.1/2/3* growing in 1/2 MS medium containing SA, BA, pHBA (10 μM, 30 μM, and 50 μM) for 10 days. (**B**) The root length of WT and triple mutant btb-a2.1/2/3 growing in 1/2 MS medium containing SA, BA, *p*HBA (10 μM, 30 μM, and 50 μM) for 10 days. (**C**, **E**) The phenotypic analysis of WT and triple mutant *btb-a2.1/2/3* growing in1/2 MS medium containing 10 μM ACC, 50 μM ACC and 10 μM AgNO3 in dark for 4 days (**C**) and in light for 7 days (**E**). (**D**) The statistics of the hypocotyl length in dark. (**F**) The statistics of the root length analysis in light. Data are mean ± SD. Values labeled with different letters are significantly different ($p<0.05$).

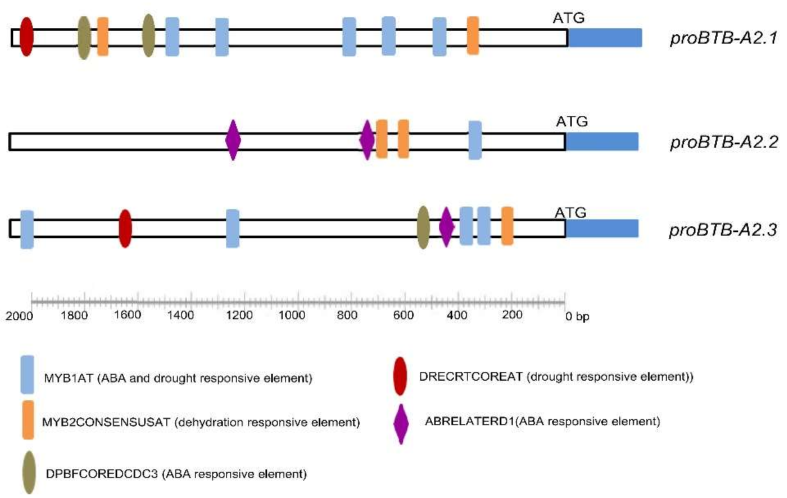

Figure S6. Analysis of *cis*-elements in the promoter of *AtBTB-A2s*. About 2000bp promoter of the BTB-A2s were respectively analyzed using *PlantCARE*.

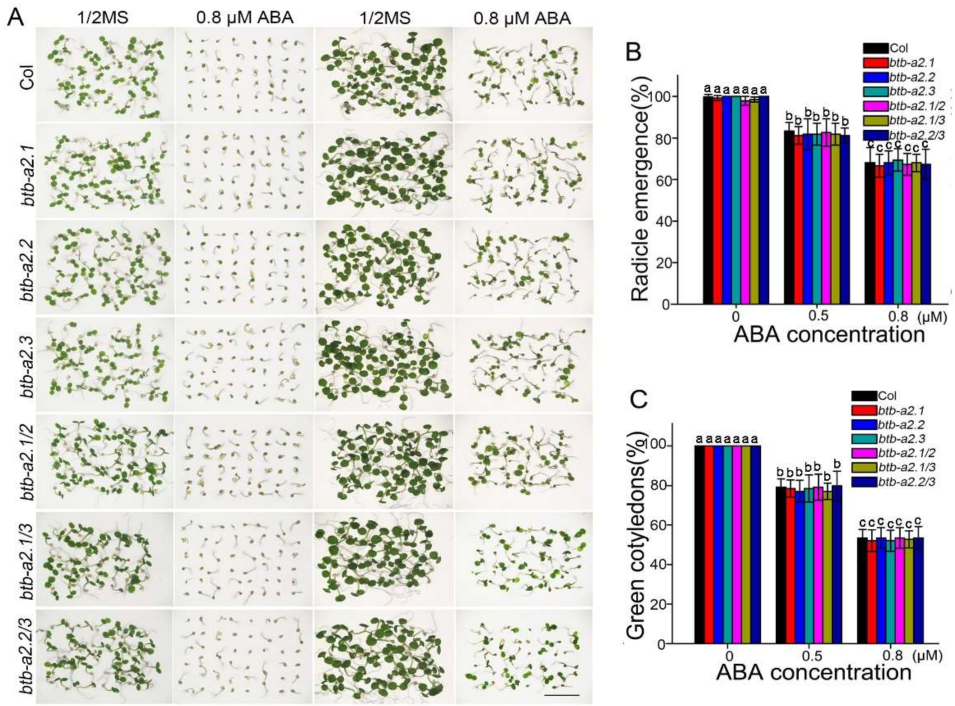

Figure S7. *Arabidopsis btb-a2* single and double mutant display no sensitivity to ABA in germination. (**A**) Germination of WT and *btb-a2.1*, *btb-a2.2*, *btb-a2.3* single and double mutants in normal and 0.8 μM ABA 1/2 MS medium. The images were taken after 3 days (first two columns) and 5 days (last two columns) of stratification, respectively. (**B**) Germination rate statistics. (**C**) Green cotyledon statistics. About 150 seeds of each line were used in each experiment. Values labeled with different letters are significantly different (p<0.05).

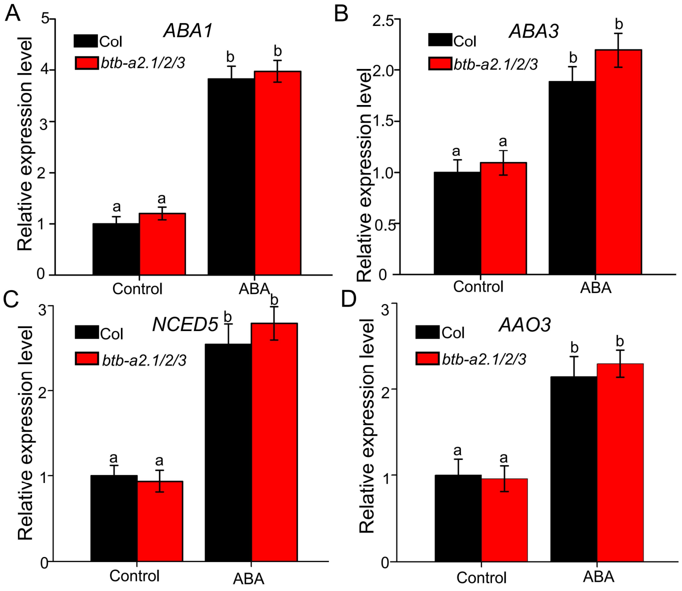

Figure S8. Expression levels of ABA synthesis related genes in WT and triple mutant *btb-a2.1/2/3*. Total RNA was isolated from 7-day-old wild-type and *btb-a2.1/2/3* seedlings growing under normal and 0.5 μM ABA conditions. *ACTIN2* gene was used as internal reference, and the results were shown by mean standard deviation. Data are mean ±SD. n=3. Values labeled with different letters are significantly different ($p<0.05$).

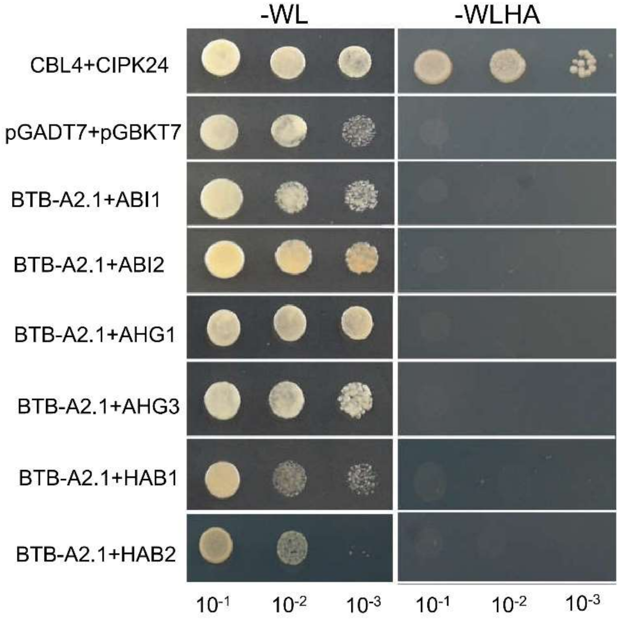

Figure S9. BTB-A2.1 may do not interact with PP2Cs. The interaction of BTB-A2.1 with ABI1, ABI2, AHG1, AHG3, HAB1, and HAB2 was performed by yeast two-hybrid assays. Saturated cultures were spotted onto on SD-WL and SD-WLHA at different dilutions (10^{-1}, 10^{-2}, and 10^{-3}).

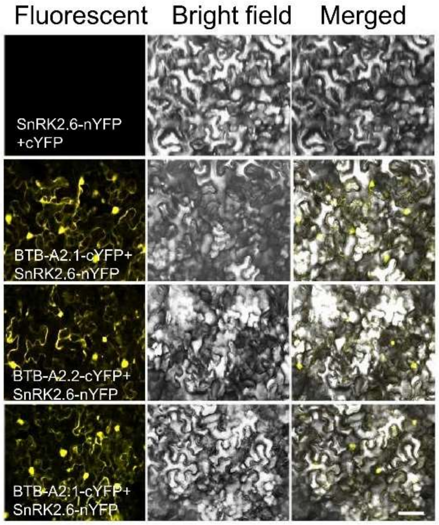

Figure S10. The interactions between BTB-A2.1, BTB-A2.2, and BTB-A2.3 with SnRK2.6 by BiFC assays in *N. benthamiana* leaves. Columns from left to right were fluorescent signal, bright field images, and merged images, respectively. Bar=50 μm.

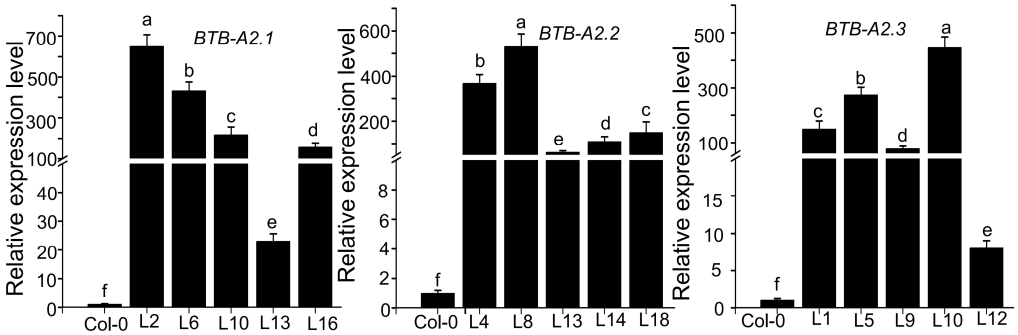

Figure S11. Expression levels of *BTB-A2.1*, *BTB-A2.2* and *BTB-A2.3* in transformed *Arabidopsis* plants by qPCR. Total RNA was isolated from 2-week-old hydroponic culture seedlings. *ACTIN2* gene was used as internal reference. Data are mean ±SD. n=3. Values labeled with different letters are significantly different ($p<0.05$).

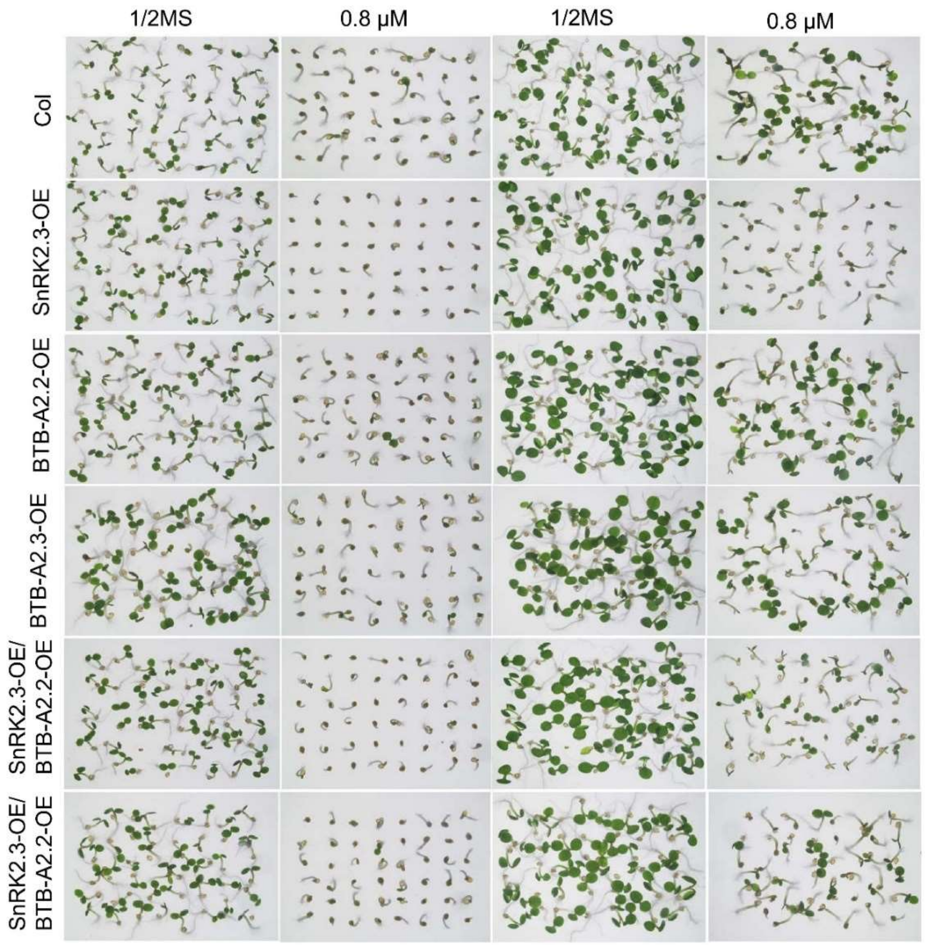

Figure S12. Overexpression of *BTB-A2.2*, *BTB-A2.3* inhibits the ABA hypersensitive phenotypes of lines overexpressing *SnRK2.3*. The seeds of WT, *SnRK2.3*-OE lines, *BTB-A2.2*-OE lines, *BTB-A2.3*-OE lines, *SnRK2.3*-OE lines in *BTB-A2.2-OE* background and *SnRK2.3*-OE lines in *BTB-A2.3*-OE background were germinated in 1/2 MS medium containing 0 and 0.8 μM ABA. The images of first two columns and last two columns were taken after 3 and 5 days of seed stratification, respectively. About 150 seeds of each line were used in each experiment, and each assay repeated 3 times.

Table S1. Primers used in this study.

Primer Name	Primer Sequences (5'-3')
LBa1	TGGTTCACGTAGTGGGCCATCG
SLLBb1	GCCTTTTCAGAAATGGATAAATAGCCTTGCTTCC
A140-Oligod(T)	TTTTTTTTTTTTTTTTTT
35S	GACGCACAATCCCACTATCC
BTB-A2.1-LP	AATGCAAACATCCTTCACAGC
BTB-A2.1-RP	ATCACTTCGTTTGATTGGTCG
BTB-A2.2-LP	ACGATATCACATCGATCTGCC
BTB-A2.2-RP	TTTCCCGCAACAATTAGTGTC
BTB-A2.3-LP	CTCCGTACGGGAGACCTTAAC
BTB-A2.3-RP	ACGTGAACAAAAGCAAACCAG
BTB-A2.1-OE-F	ACGGGGGACTCTTGACCATGGATGAATTTTCCGACGATCC
BTB-A2.1-OE-R	GTCACCTGTAATTCACACGTGGTGGTGGTGGTGGTGCTCGACG GATACCACACCGG
BTB-A2.2-OE-F	ACGGGGGACTCTTGACCATGGATGGTGGTTTCCGATGGCG
BTB-A2.2-OE-R	GTCACCTGTAATTCACACGTGGTGGTGGTGGTGGTGCTCGACG GATACCACACCGG
BTB-A2.3-OE-F	ACGGGGGACTCTTGACCATGGATGGGTATCTCAAAAGACAG

BTB-A2.3-OE-R	GTCACCTGTAATTCACACGTGGTGGTGGTGGTGGTGTATGATCG GACAAGGCGGAGT
BTB-A2.1-GFP-F	GGGGTACCATGAATTTTCCGACGATCCCTC
BTB-A2.1-GFP-R	TCCCCCCGGGGATAGATATTCCACGACTAGGAC
BTB-A2.2-GFP-F	GGGGTACCATGGTGGTTTCCGATGGCGGCAAAC
BTB-A2.2-GFP-R	TCCCCCCGGGGCTCGACGGATACCACACCGGAGAAAC
BTB-A2.3-GFP-F	CCCTCGAGATGGGTATCTCAAAAGACAG
BTB-A2.3-GFP-R	GGATCCCGTATGATCGGACAAGGCGGAGT
BTB-A2.1-GUS-F	GCTCTAGATTCGCCAAGCAAACGCAG
BTB-A2.1-GUS-R	CGGGATCCGTTCGATTCATGAGGGATCGTC
BTB-A2.2-GUS-F	TGCCTGCAGGTCGACTCTAGACAACTATATGAAAGTAAGCG
BTB-A2.2-GUS-R	ATAAGGGACTGACCACCCGGGGACACTCGTTTGCCGCCATC
BTB-A2.3-GUS-F	GCTCTAGAGACCTTGAGTCCTTGACGAT
BTB-A2.3-GUS-R	CGGGATCCTCCACCAACGTTGAATTTGAT
BTB-A2.1-EcoRI-Y2H-F	ATGGCCATGGAGGCCGAATTCATGAATTTTCCGACGATCCCT
BTB-A2.1-BamHI-Y2H-R	CCGCTGCAGGTCGACGGATCCTTAGATAGATATTCCACGAC
BTB-A2.2-EcoRI-F(YH)	CGGAATTCATGGTGGTTTCCGATGGCGGC
BTB-A2.2-XhoI-R(YH-AD)	CCCTCGAGTTACTCGACGGATACCACACCGGAG
BTB-A2.2-PstI-R(YH-BD)	AACTGCAGTTACTCGACGGATACCACACCGGAG
BTB-A2.3-EcoRI-Y2H-F	GGAATTCATGGGTATCTCAAAAGACAGGAT
BTB-A2.3-BamHI-Y2H-R	CGGGATCCTTATATGATCGGACAAGGCGGAGT
SnRK2.3-Flag-Nco-F	ATAAGATGGATCGAGCTCCGGTGAC
SnRK2.3-SpeI-R	GACTAGTTTAGAGAGCGTAAACTATCTCT
SnRK2.3-BIFC-BamHI-F	CGGGATCCATGGATCGAGCTCCGGTGACCAC
SnRK2.3-BIFC-KpnI-R	GGGGTACCGAGAGCGTAAACTATCTCT
SnRK2.3-YH-F	GGAATTCATGGATCGAGCTCCGGTGACCAC
SnRK2.3-YH-R	CGGGATCCTTAGAGAGCGTAAACTATCTCT
SnRK2.6-Flag-Nco-F	ATAAGATGGATCGACCAGCAGTGAGT
SnRK2.6-SpeI-R	GACTAGTTCACATTGCGTACACAATCTC
SnRK2.6-BIFC-BamHI-F	CGGGATCCATGGATCGACCAGCAGTGAGTGGTC
SnRK2.6-BIFC-KpnI-R	GGGGTACCCATTGCGTACACAATCTCTCCG
SnRK2.6-YH-F	GGAATTCATGGATCGACCAGCAGTGAGTGGTC
SnRK2.6-YH-R	CGGGATCCTCACATTGCGTACACAATCTCTCCG
SnRK2.2-YH-F	GGAATTCATGGATCCGGCGACTAATTCACCG
SnRK2.2-YH-R	CTGCAGTCAGAGAGCATAAACTATCTCTCCAC
BTB-A2.1-qRT-F	AGGGGAAGTTTTCTACGCCG
BTB-A2.1-qRT-R	GCTTGCATTCCCCACCAAAC
BTB-A2.2-qRT-F	GGAACCGTCCGTACACATCT
BTB-A2.2-qRT-R	TTCCGAATCAGCAACGGCG
BTB-A2.3-qRT-F	GCCAACATCCCTGAGCGTCT
BTB-A2.3-qRT-R	ATCAGGACCTGCCCTGATGGC
ABI3-qRT-F	CACAGCCAGAGTTCCTTCCTTTACT
ABI3-qRT-R	TAGTTGCTGAGGAACACAAACGG
ABI4-qRT-F	GGGCAGGAACAAGGAGGAAGTG
ABI4-qRT-R	TCTCCTCCAAAAGGCCAAATGGT
ABI5-qRT-F	ATGATCAAGAACCGCGAGTCTGC
ABI5-qRT-R	CGGTTGTGCCCTTGACTTCAAAC
RAB18-qRT-F	GGCTTGGGAGGAATGCTTCA
RAB18-qRT-R	CGCTTGAGCTTGACCAGACT
RD29A-qRT-F	GGAAGTGAAAGGAGGAGGAGGAA
RD29A-qRT-R	CACCACCAAACCAGCCAGATG
RD29B-qRT-F	GAATCAAAAGCTGGGATGGA
RD29B-qRT-R	TGCTCTGTGTAGGTGCTTGG
ABA1-qRT-F	CGTGCGGTTGGAGAAGATGTGAT
ABA1-qRT -R	TCTCAGAATGGCTTCCTCCTCAGT
ABA3-qPCR-F	AGTGGATATTGAAGAGGCAGC
ABA3-qPCR-R	CACCAGATCTAGATTAAACCTCAGG
AAO3-qRT -F	CAACCGCATGCGCACTAG
AAO3-qRT -R	GTCTTGCGGTTCAAAAACATCTT

NCED3-qPCR-F	GAGTGTCCTGTCTGAAATCCG
NCED3-qPCR-R	CGAATCCTGAGACTTTAGGCC
Actin2-F	ACTCTCCCGCTATGTATGTCGCC
Actin2-R	ATTTCCCGCTCTGCTGTTGTGGT
